# Engineering the Effector Domain of the Artificial Transcription Factor to Improve Cellulase Production by *Trichoderma reesei*

**DOI:** 10.3389/fbioe.2020.00675

**Published:** 2020-06-25

**Authors:** Qing-Shan Meng, Fei Zhang, Wei Wang, Chen-Guang Liu, Xin-Qing Zhao, Feng-Wu Bai

**Affiliations:** ^1^State Key Laboratory of Microbial Metabolism, Joint International Research Laboratory of Metabolic and Developmental Sciences, School of Life Sciences and Biotechnology, Shanghai Jiao Tong University, Shanghai, China; ^2^State Key Lab of Bioreactor Engineering, East China University of Science and Technology, Shanghai, China

**Keywords:** *Trichoderma reesei*, cellulase production, artificial transcription factors, effector domain, lignocellulosic biomass

## Abstract

Filamentous fungal strains of *Trichoderma reesei* have been widely used for cellulase production, and great effort has been devoted to enhancing their cellulase titers for the economic biorefinery of lignocellulosic biomass. In our previous studies, artificial zinc finger proteins (AZFPs) with the Gal4 effector domain were used to enhance cellulase biosynthesis in *T. reesei*, and it is of great interest to modify the AZFPs to further improve cellulase production. In this study, the endogenous activation domain from the transcription activator Xyr1 was used to replace the activation domain of Gal4 of the AZFP to explore impact on cellulase production. The cellulase producer *T. reesei* TU-6 was used as a host strain, and the engineered strains containing the Xyr1 and the Gal4 activation domains were named as *T. reesei* QS2 and *T. reesei* QS1, respectively. Compared to *T. reesei* QS1, activities of filter paper and endoglucanases in crude cellulase produced by *T. reesei* QS2 increased 24.6 and 50.4%, respectively. Real-time qPCR analysis also revealed significant up-regulation of major genes encoding cellulase in *T. reesei* QS2. Furthermore, the biomass hydrolytic performance of the cellulase was evaluated, and 83.8 and 97.9% more glucose was released during the hydrolysis of pretreated corn stover using crude enzyme produced by *T. reesei* QS2, when compared to the hydrolysis with cellulase produced by *T. reesei* QS1 and the parent strain *T. reesei* TU-6. As a result, we proved that the effector domain in the AZFPs can be optimized to construct more effective artificial transcription factors for engineering *T. reesei* to improve its cellulase production.

## Introduction

Lignocellulosic biomass is abundantly available as a renewable resource, which is mainly composed of cellulose, hemicelluloses, and lignin. Degradation of the cellulose component by cellulase into glucose to produce biofuels and biobased chemicals has attracted extensive research attention (De Bhowmick et al., 2018). However, high production cost of cellulase makes the bioconversion process too expensive for practical applications, which ultimately limits the utilization of lignocellulosic biomass for biorefinery (Klein-Marcuschamer et al., 2012).

Filamentous fungi are commonly used as cellulase producers, among which *Trichoderma reesei* has been widely studied for cellulase production (Liu et al., 2013). Without doubt, enhancing cellulase production by *T. reesei* is of great importance for developing lignocellulosic biorefinery.

The cellulase enzymatic complex of *T. reesei* has been shown to consist of at least two cellobiohydrolases (CBHs), eight endo-β-1,4-glucanases (EGs), and seven β-glucosidases (BGLs) that act synergistically upon insoluble cellulose substrate (Druzhinina and Kubicek, 2017). The synthesis of these cellulase components is strictly controlled by various regulators, including at least six positive transcriptional activators (Xyr1, Ace2, Ace3, Vib1, BglR, and the Hap2/3/5 complex) as well as three repressors (Ace1, Rce1, and the carbon catabolite repressor Cre1) (Aro et al., 2001, 2003; Cao et al., 2017; Zhang F. et al., 2018). Recently, great effort has been made to genetically modify these endogenous transcription factors to reprogram the transcriptional regulation network to improve cellulase production in *T. reesei* (Derntl et al., 2013; Lv et al., 2015; Zhang et al., 2017; Rassinger et al., 2018; Wang et al., 2019). On the other hand, artificial transcription factors (ATFs) have also been studied to genetically engineer cellulase production. For example, the repressor Cre1 could be changed to transcriptional activator for cellulases gene expression by replacement of the VP_16_ activation domain (AD) (Zhang J. et al., 2018). In the previous studies, a library of artificial zinc finger proteins (AZFPs) was explored for expression in bacteria and yeast (Park et al., 2003; Ma et al., 2015), and screened for mutants with changed phenotypes. The AZFPs were designed to be composed of four zinc fingers as a DNA-binding domain (DBD) followed by an AD of Gal4p (Gal4_AD_). We have modified the library and successfully obtained mutants with enhanced cellulase production in *T. reesei* Rut-C30 (Zhang et al., 2016). However, Gal4_AD_ in the AZFPs originates from budding yeast *Saccharomyces cerevisiae*, and it remains unknown whether exogenous transcriptional regulation domains can efficiently recruit protein complex to initiate transcription. Therefore, it is of great interest to investigate the effect of endogenous effector domains in the AZFPs on regulation of cellulase production in *T. reesei*.

In *T. reesei*, Xyr1 is the Gal4 family transcription activator that is essential for cellulase/hemicellulase gene transcription (Dos Santos Castro et al., 2016). We therefore designed a new transcription factor AZFP_M2_-Xyr1_AD_ harboring the native effector domain of Xyr1 in this study. Production of cellulase, transcription of key genes related to cellulase biosynthesis, and degradation of lignocellulosic biomass in the recombinant strain carrying AZFP_M2_-Xyr1_AD_ were compared with the parent strain and the control strain with the exogenous Gal4_AD_. The results provide basis for further optimizing the effector domains of AZFPs to enhance their stimulating effects on cellulase production by *T. reesei*.

## Materials and Methods

### Strains, Culture Media, and Culture Conditions

*Escherichia coli* GB05-dir was used for vector construction and propagation, which was cultivated in lysogeny broth (LB) medium with 4 μg/mL tetracycline (Wang et al., 2016).

*T. reesei* TU-6 (ATCC MYA-256), a non-homologous end joining pathway deficient, uridine auxotrophic derivative of QM9414 (Gruber et al., 1990), was used as the parent strain in this study. The strain and its derivatives were cultured on PDA plate for 5–7 days at 28°C to produce conidia. For fermentation experiment, *T. reesei* strains were inoculated with 10^6^ spores/mL into 250 mL Erlenmeyer flasks containing 50 mL minimal medium (MM) supplemented with 0.1% uridine, 0.2% peptone, and 2% glucose at 28°C and 200 rpm for 48 h. Then, mycelia were harvested by filtration and washed twice with MM solution without any carbon source. Equal amounts (0.4 g cell wet weight) of mycelia were transferred into 250 mL Erlenmeyer flask containing 50 mL MM supplemented with 2% (w/v) microcrystalline cellulose and 2% (w/v) wheat bran, and were cultivated at 28°C, shaking at 200 rpm. The composition of the MM solution is described in the previous report (Liu et al., 2016).

### Plasmid Construction and Fungal Transformation

Firstly, the AD of Xyr1 was amplified from *T. reesei* TU-6 cDNA and fused with the AZFP_M2_-Gal4 DBD amplified from *T. reesei* M2 genomic DNA by overlap extension PCR using primers AZFP-F and Xyr1-R, generating *Azfp*_M2_*-xyr1*_AD_ coding sequence. To overexpress the AZFPs (*Azfp*_M2_*-gal4_AD_* and *Azfp*_M2_*-xyr1*_AD_) at the *xyn3* loci in *T. reesei* TU-6, the *Azfp*_M2_*-gal4_AD_* coding sequence was amplified from *T. reesei* M2 genomic DNA, and then the two AZFPs coding sequences were ligated into the *Nco* I and *Xba* I sites of pCB303 (Zhang et al., 2016) to obtain the plasmids pCB310 and pCB311, respectively. Subsequently, the two AZFPs (AZFP_M2_-Gal4_AD_ and AZFP_M2_-Xyr1_AD_) expression cassettes which contained the AZFPs coding sequence and the terminator T*trpC* were amplified from pCB310 and pCB311, respectively. Additionally, Two DNA fragments containing approximately 1.5 kb of up- and downstream the *xyn3* non-coding region and the *pyr4* selection marker cassette were amplified from *T. reesei* QM9414 genomic DNA, respectively. Finally, five fragments including the AZFP (AZFP_M2_-Gal4_AD_ or AZFP_M2_-Xyr1_AD_) expression cassette, the *pyr4* expression cassette, the up- and downstream *xyn3* non-coding region, and the pUG6 fragment amplified from the pUG6 vector were joined into the pUG6-AZFP_M2_-Gal4_AD_ or pUG6-AZFP_M2_- Xyr1_AD_ vector by RecET direct cloning technology (Wang et al., 2016). All primers used in this study were listed in Supplementary Table S1.

The protoplast transformation protocol follows previous report (Derntl et al., 2015). Transformants were cultivated and screened on MM plates containing 2% glucose without uridine. Southern blot was performed to verify the correct mutants. *T. reesei* QS1 and QS2 strain are the engineered strains overexpression *Azfp*_M2_*-gal4_AD_* and *Azfp*_M2_*-xyr1*_AD_ at the *xyn3* loci, respectively.

#### Southern-Blot Analysis

Chromosomal DNA was isolated from mycelia by grinding in liquid nitrogen as described previously (Derntl et al., 2015). For analysis of *T. reesei* QS1 and QS2 genome, *Nde* I was used as the restriction endonuclease for genome digestion. Probes were amplified from the genomic DNA with the primers xyn3-probe-F/R in Supplementary Table S1. Then, the *Nde* I-digested genomic DNA was hybridized by the probe. Finally, the probe-hybridized DNA fragments were detected and visualized with the DIG high prime DNA Labeling and Detection Starter kit I (Roche Diagnostics, Mannheim, Germany), respectively.

### Western Blot Analysis

The conidia of the parent strain *T. reesei* TU-6 and the transformants were cultivated in MM medium containing 2% cellulose as a carbon source and 0.1% uridine to support growth. The mycelia at 72 h were collected by filtration and used to extract intracellular protein after grinding in liquid nitrogen. For detection of the His-tagged AZFP, 30 μg cell protein extracts were separated in 12% SDS-PAGE, and then the proteins were electro-transferred to PVDF membrane (Millipore, United States). An anti-His antibody (GenScript, Nanjing, China) was used to incubate with the membrane, washed, and subsequently incubated with HRP-conjugated goat anti-rabbit IgG as a secondary antibody. Bands on the blotting membrane were visualized using the DAB kit (CWBIO, Shanghai, China) according the manufacturer’s instructions.

### Biochemical Assays

The activities of filter paper (FPase), endo-β-glucanase (CMCase), exo-β-glucanase (*p*NPCase), β-glucosidase (*p*NPGase), and Xylanase were determined as described elsewhere (Wood and Bhat, 1988; Gao et al., 2017). Total extracellular proteins were assayed using the BCA Kit (Beyotime, Shanghai, China).

### Transcription Analysis by RT-qPCR

The *T. reesei* strains were cultured, and harvested at 24 and 48 h. Total RNA was extracted using the Spin Column Plant Total RNA Purification Kit (Sangon Biotech, China), and 1μg RNA was reverse transcribed to cDNA using the PrimeScript^®^ RT Reagent Kit with gDNA Eraser (Takara Japan). RT-qPCR analysis was carried out with iQ SYBR Green Supermix Kit (Bio-Rad, United States) and the CFX Connect Real-Time PCR Detection 96 System (Bio-Rad, United States) using the primers listed in Supplementary Table S2. Three biological replicates for all reactions were carried out, and the relative transcription of genes was calculated according to the 2^–ΔΔCT^ method using the reference gene *tef1* for normalization (Livak and Schmittgen, 2001; Steiger et al., 2010).

### Saccharification of Pretreated Lignocellulosic Biomass

Alkaline-pretreated corn stover (APCS) and Jerusalem artichoke stalk (APJAS) were used as substrates in the saccharification process and the chemical compositions of APCS and APJAS were described before (Meng et al., 2018). The crude enzyme was placed in 30 mL citrate buffer (50 mM, pH4.8) containing 5% (W/V) substrate and the reaction mixture was incubated at 150 rpm, 50°C. Enzyme loading was adjusted to the same protein dosage (30 mg/g substrate). Glucose released was detected by HPLC at interval of 12 h. Cellulose conversion was calculated as follows:

$$\begin{array}{c} \mathrm{Cellulose}\mathrm{conversion}\;\text{=}\;\frac{\mathrm{Glucose}\mathrm{yields}\;\left(\text{mg}\right)}{\mathrm{Substrate}\mathrm{weight}\;\left(\text{mg}\right)\times \mathrm{Cellulose}\mathrm{content}\;\left(\%\right)}\;\times \;0.9\;\times \;100\% \end{array}$$

## Results

### Construction of the Recombinant *T. reesei* Containing the Novel Artificial Transcription Factor AZFP_M2_-Xyr1_AD_

**FIGURE 1:**
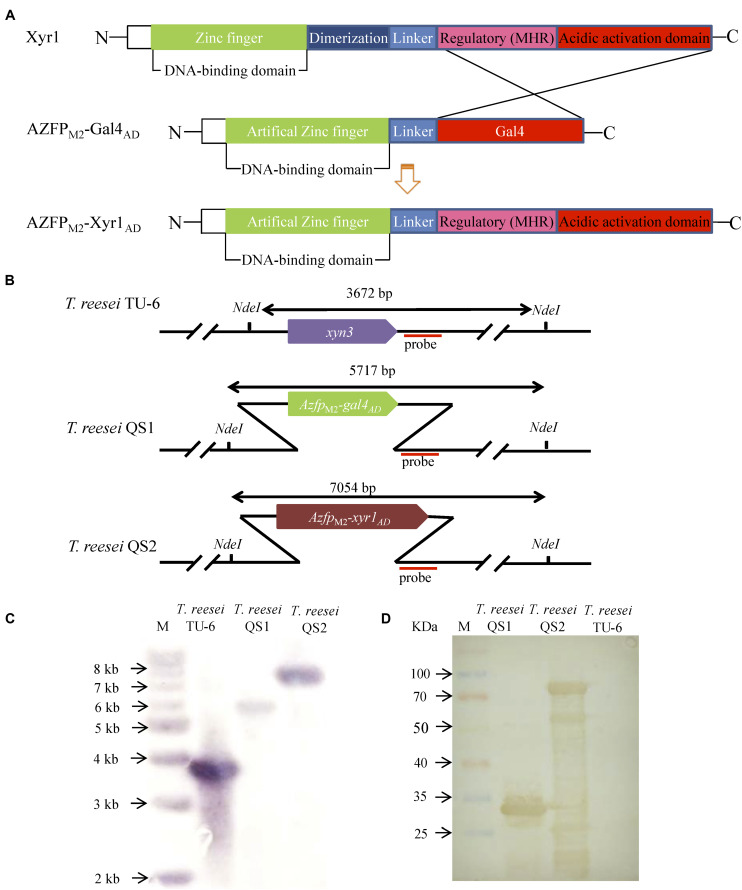
Construction of fusion transcription factor AZFP_M2_-Xyr1_AD_ in *T. reesei*. **(A)** Schematic map of AZFP_M2_-Xyr1_AD_. **(B)** Schematic diagram of Southern bolt analysis. **(C)** Southern blot analysis of transformants QS1 and QS2 with AZFPs at *xyn3* loci, respectively. Southern blot analysis of the genome digested with *Nde* I. A fragment of 3.7 kb is present in the parent strain, and the 5.7 and 7.1 kb bands are shown in the mutant strains QS1 and QS2, respectively. **(D)** Western blot analysis for AZFP_M2_-Gal4_AD_/Xyr1_AD_ in *T. reesei* QS1 and QS2, respectively. *T. reesei* TU-6 represent the parent strain.

In our previous work, transformants containing AZFPs coding sequences driven by the constitutive promoter *pki* were screened (Zhang et al., 2016), and one hyper-cellulolytic mutant *T. reesei* M2 was selected by detecting FPase activity during liquid fermentation for cellulase production in flasks (Meng et al., 2020). Subsequently, the AZFP coding sequence in *T. reesei* M2 was amplified and analyzed. As shown in Supplementary Figure S1, the AZFP coding sequence is composed of four zinc fingers acting as a DBD, followed by a Gal4_AD_, and was named AZFP_M2_-Gal4_AD_. Considering the heterologous origin of Gal4_AD_, we are interested in whether cellulase production could be further improved when Gal4_AD_ was replaced by the endogenous Xyr1_AD_. In addition, the *pki* promoter was changed to a relatively stronger promoter of *xyn3*. Therefore, a new artificial transcription factor AZFP_M2_-Xyr1_AD_ was constructed, and was constitutively expressed at the *xyn3* locus in *T. reesei* TU-6, yielding the strain *T. reesei* QS2. For comparison purposes, the AZFP_M2_-Gal4_AD_ was also inserted in the same locus, and the resultant strain was named *T. reesei* QS1 (Figure 1A). Southern blot analysis confirmed correct integration at the *xyn3* locus in *T. reesei* QS1 and QS2 strains, respectively (Figures 1B,C). On the other hand, the expression of AZFPs in the cell lysate of *T. reesei* QS1 and QS2 mutants was confirmed by Western blot analysis (Figure 1D), indicating the observed phenotypic changes of transformants in cellulase production were resulted from the expression of the integrated AZFPs.

### Influence of AZFP_M2_-Xyr1_AD_ Overexpression on Cellulolytic Activity

**FIGURE 2:**
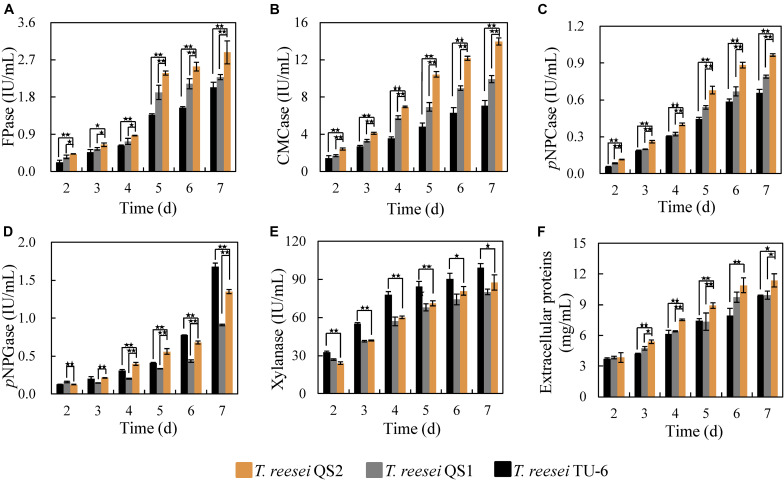
Cellulase production by *T. reesei* QS2, QS1 and TU-6. **(A–E)** The activities for FPase, CMCase, *p*NPCase, *p*NPGase and xylanase, respectively. **(F)** Total extracellular proteins content. The strains were cultured at 28°C and 180 rpm in flasks using minimal medium supplemented with 2% cellulose and 2% wheat bran as carbon source. Error bar denotes the standard deviations (SD) of three replicates (*p < 0.05, **p < 0.01).

The corresponding activities of cellulolytic enzymes from *T. reesei* QS2, QS1, and the parent strain *T. reesei* TU-6 were further evaluated. As shown in Figure 2A, *T. reesei* QS2 displayed the highest cellulase activity (FPase) of 2.4 IU/mL at the 5th day among these strains, 24.2 and 73.7% higher than that secreted by *T. reese*i QS1 and the parent strain TU-6, respectively. In addition, the endoglucanase activity (CMCase) of *T. reesei* QS2 improved 50.4 and 132.4%, respectively, compared to that of *T. reesei* QS1 and TU-6 strain (Figure 2B). In case of exoglucanase activity, *T. reesei* QS2 produced a *p*NPCase activity of 0.68 IU/mL, which displayed a 25.8 and 52.0% increase compared to the respective strains. However, we found that the activity of β-glucosidase (*p*NPGase) varied among these strains (Figure 2C). Surprisingly, *T. reesei* QS2 exhibited an obviously 48.4% higher β-glucosidase activity than *T. reesei* QS1 mutant, while 24.4% lower β-glucosidase activity than that of the parent strain TU-6 at the 7th day fermentation in flask (Figure 2D). To compare the roles of the two AZFPs in regulation of cellulase synthesis, the *xyn3* gene locus was chosen for insertion of the AZFPs. As expected, xylanase produced by *T. reesei* QS2 was similar to that of *T. reesei* QS1, but lower than that of the parent strain (Figure 2E), which was caused by the disruption of *xyn3* ORF in these two mutants. However, the extracellular proteins secreted by *T. reesei* QS2 showed a 21.5 and 20.2% increase in comparison with that of *T. reesei* QS1 and TU-6 strain, respectively (Figure 2F). These results suggest that optimization of artificial transcription factors with endogenous ADs is a viable strategy for increasing cellulase production.

### Influence of AZFP_M2_-Xyr1_AD_ Overexpression on Transcript Levels of Cellulase Encoding Genes

**FIGURE 3:**
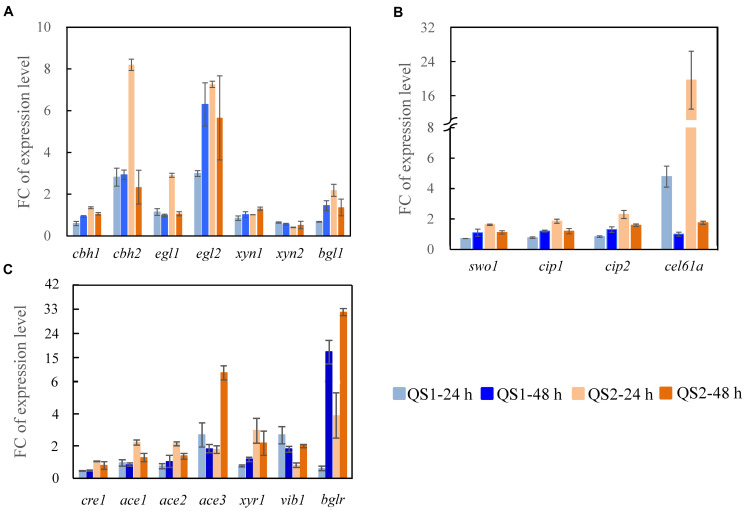
Transcription analysis of genes encoding major cellulolytic enzymes and regulators. Genes encoding **(A)** cellulolytic enzymes, **(B)** regulators, and **(C)** accessory proteins were analyzed for *T. reesei* QS1 and QS2 mutants with *T. reesei* TU-6 as the reference strain. Strains were cultured at 28°C and 180 rpm in flasks using minimal medium supplemented with 2% cellulose and 2% wheat bran as a carbon source for 24 and 48 h, respectively. Expression levels of the reference gene *tef1* were used as an endogenous control. The value is the mean of three biological replicates with SD as the error bars. FC represents fold change of the transcription levels with targeted genes detected in the mutants over that detected in the control.

To gain further insight into how the overexpression of AZFP_M2_-Xyr1_AD_ influences the regulation of the cellulase biosynthesis in *T. reesei* QS2, the transcriptional levels of major cellulase and related transcription factor genes were measured. For *T. reesei* QS2, higher expression of the major cellulase genes (*cbh1*, *cbh2*, *egl1*, *egl2*, and *bgl1*), 2. 3-, 2. 9-, 2. 5-, 2. 4-, and 3.2-folds compared with that of *T. reesei* QS1, was observed early at 24 h, which may consequently lead to more efficient production of cellulase characterized by the increased FPase activity. In addition, the expression of cellulolytic enzyme genes (*cbh2*, *egl1*, *egl2*, and *bgl1*) was also significantly up-regulated and a dramatic down-regulation of *xyn2* at 24 h was observed in *T. reesei* QS2 compared with the parent strain *T. reesei* TU-6, which was in accordance with the activities of cellulolytic enzymes produced by *T. reesei* QS2 (Figure 3A). On the other hand, the transcription of accessory protein encoding genes in *T. reesei* QS2 also exhibited significant elevation when compared with *T. reesei* QS1 and the parent strain (Figure 3B). Among these genes, the transcription of *cel61a* and *cip2* in *T. reesei* QS2 were 4.1- and 2.7-fold as well as 19.7- and 2.3-fold higher than that from *T. reesei* QS1 and TU-6 at 24 h, respectively, suggesting that the crude enzyme secreted by *T. reesei* QS2 is more suitable for further hydrolysis of lignocellulosic biomass.

In addition to the enzyme-encoding genes mentioned above, the transcriptional levels of the most known cellulase transcription factor genes were also significantly altered in both *T. reesei* QS1 and QS2 (Figure 3C). Among these transcription factor genes, *xyr1*, which encodes the major transcriptional activator for cellulase biosynthesis (Stricker et al., 2006), was significantly up-regulated by 3.8- and 2.9- fold in *T. reesei* QS2 at 24 compared with that of *T. reesei* QS1 and the parent strain, respectively. Similar trend was observed at 48 h. Even more, an enhanced expression of another transcription activator gene *ace3*, approximately 5.0- and 9.2-folds in *T. reesei* QS2 compared with that of *T. reesei* QS1 and the parent strain, was also observed at 48 h, indicating that the production of cellulase might be specifically regulated by these transcription factors. As for *bglr* encoding an activator specific for regulating β-glucosidases except *bgl1*, its expression in *T. reesei* QS2 was substantially enhanced to 6.5-folds at the early stage of 24 h, and then attenuated to 1.9-folds at 48 h compared with that of *T. reesei* QS1. Whereas, *T. reesei* QS2 and QS1 showed highly similar transcription profile across the tested genes in comparison to the parent strain, but the magnitude of gene expression change was more pronounced in *T. reesei* QS2. Conclusively, we assume that the AZFP_M2_-Xyr1_AD_ is more advantageous for regulation of cellulase biosynthesis in *T. reesei* than the AZFP_M2_-Gal4_AD_.

### Saccharification of Lignocellulosic Substrates by Cellulolytic Enzyme Preparations From the *T. reesei* Strains

**FIGURE 4:**
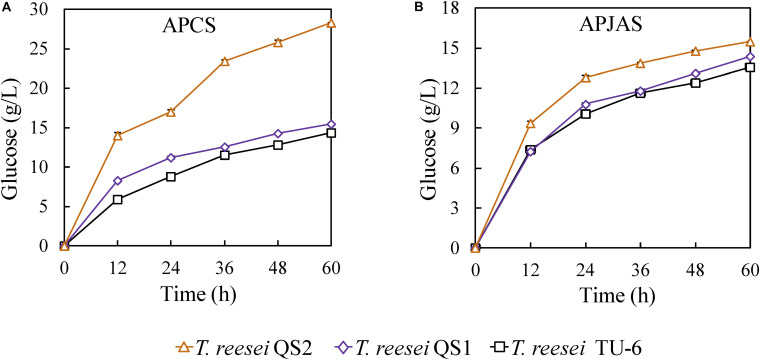
Hydrolysis of alkali pretreated corn stover (APCS) and jerusalem artichoke stalk (APJAS) by cellulases produced by *T. reesei* QS2, QS1, and TU-6. Sugar release from **(A)** alkali pretreated corn stover (APCS) and **(B)** alkali pretreated jerusalem artichoke stalk (APJAS) was examined. The same protein dosage (30 mg/g substrate) was used for enzymatic hydrolysis experiments. Data are represented as the mean of triplicate with standard deviation (SD) as the error bars.

In order to evaluate the hydrolysis ability of the cellulase produced by the *T. reesei* strains on lignocellulosic biomass, the crude enzyme complexes were used to saccharify APCS and APJAS. At the same enzyme loading (30 mg/g substrate), the glucose release (28.3 g/L corresponding to 86.9% cellulose conversion) using the *T. reesei* QS2 enzyme in the saccharification of APCS increased 83.8 and 97.9% compared with that from *T. reesei* QS1 (15.4 g/L corresponding to 47.3% cellulose conversion) and TU-6 (14.3 g/L corresponding to 43.9% cellulose conversion) at 60 h (Figure 4A). In contrast, similar as described in the previous reports, less glucose was released using APJAS as a substrate, and less difference was observed using crude enzymes from different strains, which would be caused by different composition and structure of APCS and APJAS (Meng et al., 2018). However, we still found slightly more glucose release by cellulase produced with *T. reesei* QS2 among the three strains. The glucose concentrations are 15.5, 14.4, and 13.6 g/L (corresponding to 58.7, 54.5, and 51.4% cellulose conversion), for *T. reesei* QS2, QS1 and the parent strain, respectively (Figure 4B). These results revealed that the optimized enzyme system of *T. reesei* QS2 benefits scarification of lignocellulosic biomass.

## Discussion

Based on the existing knowledge about the regulatory network of cellulase biosynthesis in *T. reesei*, great efforts have been made to reprogram cellulase transcription with the aim to enhance cellulase production by engineering endogenous transcription factors. However, the regulatory mechanism of cellulase biosynthesis is still not fully clear, and the cross-regulation of transcription factors involved in cellulase biosynthesis is very complicated, which all limit the development of robust strains (Liu and Qu, 2019). Taking advantage of the diversity of zinc finger DBDs, AZFPs with the yeast Gal4_AD_ have been developed to increase cellulase production in *T. reesei* (Zhang et al., 2016). Yeast Gal4p has been identified as a transcription activator for regulation of genes involved in galactose catabolims, which contains an N-terminal DBD (1∼147aa) and a C-terminal AD (768∼881aa) (Hidalgo et al., 2001). The acidic part of the C-terminal activation region functions to stimulate transcription via the recruitment of SAGA (Spt-Ada-Gcn5-Acetyltransferase) *in vivo* (Larschan and Winston, 2001). The yeast Gcn5 orthologous protein encoding gene in *T. reesei* has also been identified, which plays a crucial role in regulation of cellulase gene expression (Xin et al., 2013). However, no further evidences showed that the exogenous Gal4_AD_ activates transcription by recruiting SAGA complex for the regulation of cellulase biosynthesis in *T. reesei* at present. Xyr1 (Xylanase regulator) is a Gal4-like binuclear Zn-cluster protein, which can activate the expression of cellulase and hemicellulase gene expression in *T. reesei*. Lack of Xyr1 resulted in not only the absence of lignocellulosic enzyme production, but also down-regulation of genes encoding MFS (Major facilitator superfamily) transporters and ABC (Adenosine triphosphate (ATP)-binding cassette) transporters in inducing medium (Dos Santos Castro et al., 2016). As shown in Figure 1A, the highly conserved domain of this protein contains the DBD (91∼131aa) recognizing the nucleotide binding motif [GGC(T/A)_4_] (Furukawa et al., 2009), as well as a middle homology region (MHR, 314∼632aa) and a short acidic AD (767∼940aa) within the distal portion of the C-terminus. The missing 140 C-terminal amino acids of Xyr1 abolished cellulase production (Lichius et al., 2015). Recent studies have revealed the transcription factor Xyr1_AD_ could recruit SWI/SNF to remodel nucleosomes positioned in cellulase gene promoters for activating their transcription (Cao et al., 2019), suggesting the important role of this AD in regulation of cellulase gene expression. Therefore, we speculated that Xyr1_AD_ may function better than Gal4_AD_ to regulate cellulase gene expression in *T. reesei*. Moreover, it was reported that the mating type locus protein MAT1-2-1 could directly interact with the MHR and AD of Xyr1 to regulate expression of cellulase genes in response to light, which suggest that the MHR might be critical for enhancing transcription of target genes (Zheng et al., 2017). Herein, we used the endogenous Xyr1_AD_ including MHR instead of the original Gal4_AD_ to rebuild a new AZFP, and the results demonstrated that this optimization of AZFP not only improves cellulase production, but also balances cellulase system for efficient biomass conversion. In our recent work, it was found that the AZFP_M2_-Gal4_AD_ could down-regulate the expression of a newly identified cellulase repressor Ctf1, resulting in relief of the repression of cellulase activator Vib-1 and Ace3 by Ctf1 for improvement of cellulase production (Meng et al., 2020), which was consistent with transcription analysis in Figure 3C (Fold change ¿ 2). Here, the expression of *ctf1* was also significantly down-regulated by AZFP_M2_-Gal4_AD_, but not by AZFP_M2_-Xyr1_AD_ (Supplementary Figure S2), which suggest that the regulatory mechanisms of these two AZFPs on cellulase biosynthesis are different due to the different effector domains. Taken together, our results proved the important roles of the effector domains in the AZFP regulating cellulase production in filamentous fungi. Using synthetic biology tools, other effector domains as well as synthetic effector domains can be created, and the numbers of effector domains can also be modulated in the AZFPs to improve cellulase production.

In order to compare the roles of the AZFP_M2_-Gal4_AD_ and AZFP_M2_-Xyr1_AD_ in cellulase biosynthesis, the *xyn3* gene locus was chosen for foreign DNA insertion, since the *xyn3* promoter was optimal for the expression of a specific gene and deletion of *xyn3* gene did not affect the cellulase activity (Rahman et al., 2009). The results showed that the activities of CMCase and *p*NPCase were improved significantly, which was consistent with transcription analysis in Figure 3. In the future work, various other integration sites can be evaluated to optimize the cellulase enzymatic complex. The cellulase produced by *T. reesei* QS2 displayed better performance in saccharification of lignocellulosic biomass comparing with that of *T. reesei* QS1. Besides the increased β-glucosidase activity in cellulase mixture of *T. reesei* QS2 in comparison to that of *T. reesei* QS1 (Figure 2D), another possible reason for enhanced hydrolysis efficiency is the enhancement of accessary protein components, such as Swo1, Cip1, Cip2, and Cel61A, the encoding genes of which were up-regulated in *T. reesei* QS2 (Figure 3). The protein Cip2 exhibits synergistic activity with the lytic polysaccharide monooxygenase (Cel61A) to break down the linkage present in the hemicellulose-lignin matrix (Duranová et al., 2009; Pierce et al., 2017). It will be important to explore regulation of accessory protein biosynthesis by the AZFP developed in this study, so that further synthetic biology design can be performed to increase hydrolysis efficiency of lignocellulosic biomass.

## Conclusion

In this study, we designed a new artificial transcription factor AZFP_M2_-Xyr1_AD_ in *T. reesei*, and found that AZFP_M2_-Xyr1_AD_ could significantly improve the expression of major cellulase genes. Moreover, the cellulase produced by the *T. reesei* QS2 carrying AZFP_M2_-Xyr1_AD_ was proven to be highly effective in the saccharification of the pretreated lignocellulosic biomass. As a result, we proved that the endogenous effector domain Xyr1_AD_ is more advantageous for construction of AZFP not only in improving cellulase production, but also in optimizing cellulase complex for biomass conversion.

## Data Availability Statement

All datasets generated for this study are included in the article/Supplementary Material.

## Author Contributions

X-QZ and Q-SM conceived the project and designed the experiments. Q-SM carried out experiments and measurements, and interpreted experimental data. WW, FZ, C-GL, X-QZ, and F-WB revised the manuscript. X-QZ supported the research funding. All authors read and approved the final manuscript.

## Conflict of Interest

The authors declare that the research was conducted in the absence of any commercial or financial relationships that could be construed as a potential conflict of interest.
