# Supplementary material for: Engineering the Effector Domain of the Artificial Transcription Factor to Improve Cellulase Production by Trichoderma reesei
Source: Front Bioeng Biotechnol. 2020 Jun 25;8:675. doi: 10.3389/fbioe.2020.00675 (PMC7330100; doi:10.3389/fbioe.2020.00675)
Supplement: Supplementary file 1 [file Data_Sheet_1.pdf]

Supplementary materials for the manuscript submitted to Frontiers in Bioengineering and Biotechnology

Engineering the effector domain of the artificial zinc finger transcription factor to improve cellulase production by *Trichoderma reesei*

Qing-Shan Meng[1], Fei Zhang[1], Wei Wang[2], Chen-Guang Liu[1], Xin-Qing Zhao[1*], Feng-Wu Bai[1]

[1]State Key Laboratory of Microbial Metabolism, Joint International Research Laboratory of Metabolic & Developmental Sciences and School of Life Science and Biotechnology, Shanghai Jiao Tong University, Shanghai 200240, China.

[2]State Key Lab of Bioreactor Engineering, East China University of Science and Technology, Shanghai 200237, China.

*Corresponding authors:

E-mail addresses: xqzhao@sjtu.edu.cn (X.Q. Zhao)

Supporting tables

Table S1 List of primers used in this work *

Primer name	Sequence (5'-3')	Purpose
pUG6-F	cttctcgtgcttgcagaccaggttcggtgatatcagatc cactagtggcc	Amplification of pUG6 fragment
pUG6-R	caacgcacaccacaaatggatcccggttcccgcgttct atagtgtcacct	
xyn3-upstream-F	aggtgacactatagaacgcgggaaccgggatccattt gtggtgtgcgttg	Amplification of xyn3-upstream
xyn3 upstream-R	gatggatgattgtaccccagctgcgatgcattggcctta attaaattgtc	
pyr4-F	gacaatttaattaaggccaatgcatcgcagctggggta caatcatccatc	Amplification of *pyr4* gene
pyr4-R	catatacttgactctagagataccgcgtctccctcattac taccctctcg	
xyn3-downstream-F	cgagagggtagtaatgagggagacgcggtatctctag agtcaagtatatg	Amplification of xyn3-downstream
xyn3 downstream-R	ggccactagtggatctgatatcaccgaacctggtctgc aagcacgagaag	
AZFP-F	atgcaccatcatcatcatcatcaagctatgggtgctcct cc	Amplification of $AZFP_{M2}$-Gal4 DBD
AZFP-R	cgccaaagacacggggagagcagccgctttttcacc ggtgt	
xyr1- overlap-F	tgctctccccgtgtctttggcgtg	Amplification of $Xyr1_{AD}$
xyr1-R	acgttaagtggatcctctagattagagggccagaccg gttc	
xyn3-AZFP-inf-F	caattgaggcggacaatttaatgcaccatcatcatcatc atcaagctatgggtgctcctcc	Amplification of AZFPs expression cassette
xyn3-TtrpC-inf-R	ttgtaccccagctgcgatgcatgagtggagatgtggag tgggcgc	
xyn3-protoplast-F	gagtgccggctaaacaagttcatg	Fragment amplification for protoplast transformation
xyn3-protoplast-R	tggtctgcaagcacgagaagc	
Xyn3-probe-F	aacctccctacaagcatccac	Amplification of probe fragment for southern blot verification
Xyn3-probe-F	gaacgggctgacaacatcg	

Table S2. Primers for the RT-qPCR analysis

Primer	Sequence (5’-3’)
cre1-F	CCTTACTTTGGCCAGGGTGT
cre1-R	AGTTGGGCCTTGACCTCTTG
ace1-F	ACCAAGACCAACGGCAAGA
ace1-R	CGTGGAGGAAGGCGTAGACA
ace2-F	GCCTCAATGCTGCTCTCTGTT
ace2-R	GACGAACGACCTTTGCTTCTCT
ace3-F	ATTGTGCGAGACATGCTGAG
ace3-R	GATGGCCAGCAAACTAGCTC
xyr1-F	ACAGTGGAGCGGTAACAGACA
xyr1-R	CACGAATCCTTCCGACGAG
vib-1-F	TGACCTGCTACCGAAGAAACC
vib-1-R	CCACGGGATGACAATAAGACG
bglr-F	GCAAGGTCAAGTGCGATGG
bglr-R	CTGTTGATGCGGTTGTGGA
ctf1-F	TCAACCAAAAGCCAAAGGAG
ctf1-R	GGGTCAAAGTCGGTGTGTG
cbh1-F	ACGAGTTCTCTTTCGATGTTGATG
cbh1-R	CGGTGTTGGTGGGATACTTG
cbh2-F	TCCTGGTTATTGAGCCTGAC
cbh2-R	GCAACATTTGGAAGGTTCAG
egl1-F	CTCAGATGGACGAGAACGGG
egl1-R	CTGGTGGCTAGTGTTGAGGG
egl2-F	AACAAGTCCGTGGCTCCATT
egl2-R	TCCGCTCCAACCAATACCTC
xyn1-F	AAACTACCAAACTGGCGG
xyn1-R	TTGATGGGAGCAGAAGATCC
xyn2-F	CGGCTACTTCTACTCGTACTG
xyn2-R	TTGATGACCTTGTTCTTGGTG
bgl1-F	CCGAGTGATCTGTTCCAGAATGT
bgl1-R	CTGGGTGCTGAAGATGGGTAG
CIP1-F	TCCACCGTCACTCTGCCTAC
CIP1-R	CCAGCGTCGTTTGGATTG
CIP2-F	CGCAAGAATAGACACCACCAAG
CIP2-R	AAATCCTCCAGCACGCAGA
Cel61a-F	TCAACTACATCATCCCTGGACCT
Cel61a-R	CCGTTGTCGTGGTTCTGCT

Swo1-F	GCTTCCACCTACACAACCACA
Swo1-R	TGGGCAGCAAACATTATCCA
tef1α-F	CTGGGTGTCAAGCAGCTCA
tef1α-R	GAGATGGGGACGAAAGCAAC

Supporting figures

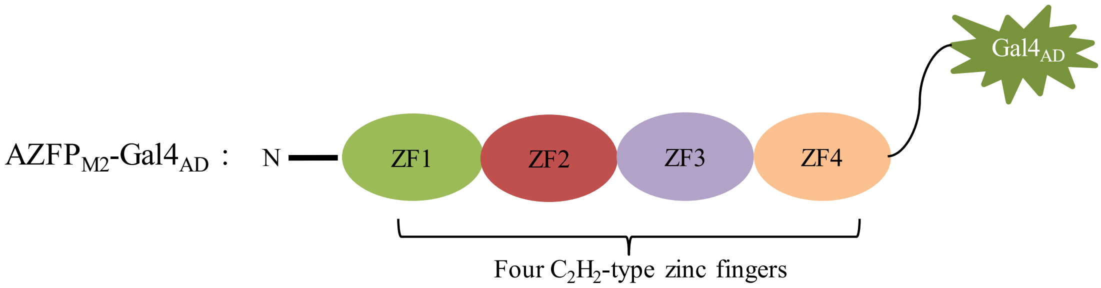

Figure S1 Schematic diagram of the AZFP$_{M2}$-Gal4$_{AD}$ in *T. reesei* M2. ZF represent a C_2H_2-type zinc finger.

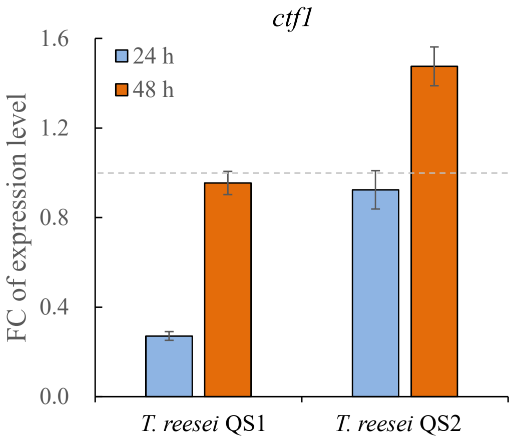

Figure S2 Gene expression of cellulase repressor encoding gene *ctf1* analyzed by quantitative RT-PCR in *T. reesei* QS1, QS2 and the parent strain TU-6. Strains were cultured at 28 °C and 180 rpm in flasks using minimal medium supplemented with 2% cellulose and 2% wheat bran as a carbon source for 24 h and 48 h, respectively. Expression levels of the reference gene *tef1* were used as an endogenous control. The value is the mean of three biological replicates with SD as the error bars. FC represents fold change of the transcription levels with *ctf1* detected in the mutants over that detected in the control.
